# Effect of lateral decubitus acquisition in accuracy and lung severity estimation of chest computed tomography in children with suspected COVID-19

**DOI:** 10.31744/einstein_journal/2022AO0061

**Published:** 2022-07-18

**Authors:** André Vaz, Bruno Maurício Pedrazzani, Jorge Alberto Ledesma, Akemi Yagui, Hugo Reuters Schelin

**Affiliations:** 1 Hospital Pequeno Príncipe Curitiba PR Brazil Hospital Pequeno Príncipe, Curitiba, PR, Brazil.; 2 Faculdades Pequeno Príncipe Instituto de Pesquisa Pelé Pequeno Príncipe Curitiba PR Brazil Faculdades Pequeno Príncipe, Instituto de Pesquisa Pelé Pequeno Príncipe, Curitiba, PR, Brazil.

**Keywords:** Coronavirus infections, COVID-19, Multidetector computed tomography, Child, Reverse transcriptase polymerase chain reaction, Tomography, X-ray computed

## Abstract

**Objective:**

To compare inter-rater reliability, diagnostic accuracy, and extension of pulmonary involvement in children with suspected COVID-19 submitted to supine or supine and lateral decubitus computed tomography imaging.

**Methods:**

Retrospective study carried out between March 2020 and March 2021 with patients submitted to reverse transcription-polymerase chain reaction testing and chest computed tomography. Patients were divided into two groups: supine or supine and lateral decubitus imaging. Standardized reporting systems of computed tomographic findings in COVID-19 and chest computed tomography score were used.

**Results:**

One hundred and seventeen patients were enrolled. Moderate to substantial inter-rater reliability was observed for standardized reporting systems (weighted kappa, 0.553-0.764; p<0.001). Inter-rater reliability for the chest computed tomography score was substantial (weighted kappa, 0.620-0.670; p<0.001). Standardized reporting systems failed to predict COVID-19 in children, regardless of additional lateral decubitus imaging (area under the receiver operating characteristic curve, 0.491-0.608). Chest computed tomography scores assigned to lateral decubitus images were significantly lower.

**Conclusion:**

Additional lateral decubitus imaging does not improve the accuracy of standardized reporting systems of computed tomographic findings in COVID-19 but may provide a more accurate estimation of lung involvement in uncooperative patients.

## INTRODUCTION

Imaging assessment of coronavirus disease 2019 (COVID-19) in children remains a challenge. In adults with COVID-19, the typical chest computed tomography (CT) finding is ground-glass opacity with peripheral distribution, especially in the lower lobes, with or without consolidation.^(1-4)^ However, children may have no CT abnormalities or discrete CT findings, heterogeneous and nonspecific lung abnormalities, or more severe pulmonary involvement in cases with superimposed lung infection.^(2,4-10)^ In non-cooperative pediatric patients with suspected COVID-19, respiratory artifacts further complicate the interpretation of chest CT images, since they may simulate ground-glass opacities and falsely increase the extent of pulmonary involvement. There are two alternatives to overcome this issue: to sedate such patients and acquire images at the end of full inspiration or to acquire additional images in lateral decubitus.^(11-13)^

Despite the countless publications on COVID-19, the impact of chest CT techniques on diagnostic accuracy, inter-rater agreement, lung lesion extension estimation and radiation exposure in children has seldom been discussed, if at all.

## OBJECTIVE

To compare inter-rater reliability, diagnostic accuracy and extension of pulmonary involvement in children with suspected COVID-19 submitted to supine or supine and lateral decubitus computed tomography imaging.

## METHODS

### Patient population

A retrospective study with children submitted to unenhanced chest CT due to suspected COVID-19 was carried out at a large-scale tertiary children’s hospital between March 2020 and March 2021. During that time, 2,977 patients with suspected COVID-19 were admitted. Of these, 629 tested positive for severe acute respiratory syndrome coronavirus 2 (SARS-CoV-2) based on ribonucleic acid identification using the reverse transcription-polymerase chain reaction (RT-PCR) assay. Children with suspected COVID-19 and moderate to severe clinical course or symptomatic deterioration, who were submitted to chest CT for complication detection or alternative diagnosis, were consecutively included. Patients older than 18 years and patients with incomplete or missing medical history or missing RT-PCR results were excluded. Follow-up scans were not used.

### Imaging technique

All imaging exams were performed using a low-dose protocol and a 64-channel multidetector CT scanner (Revolution EVO, GE Healthcare), with slice thickness of 3.75-5mm, tube voltage of 80-120kVp, automatic tube current modulation (50-80mA), rotation time of 0.4-0.6 seconds, pitch of 0.98 and Adaptive Statistical Iterative Reconstruction (ASiR)-V (total average estimated effective dose <3mSv). Patients were scanned in the supine position, from the lung apex to the diaphragm. In cooperative patients, images were acquired only in the supine position (Supine Chest CT Group). In uncooperative patients, additional right and left lateral decubitus images were acquired (Lateral Decubitus Chest CT Group). Lateral decubitus and supine image acquisitions were programmed with the same scouts. Therefore, volume loss may have occurred in small areas of the lung apex or base while rotating the patient (no more than 3/4 of lung volume lost in lateral decubitus image acquisition). The hospital’s routine approach in uncooperative patients submitted to chest CT consists of additional image acquisition in lateral decubitus position instead of sedation due to cost constraints, risks inherent to anesthesia and absence of evidence of harm of low-dose (<10mSv) CT protocols.^(14-16)^

**Figure 1:**
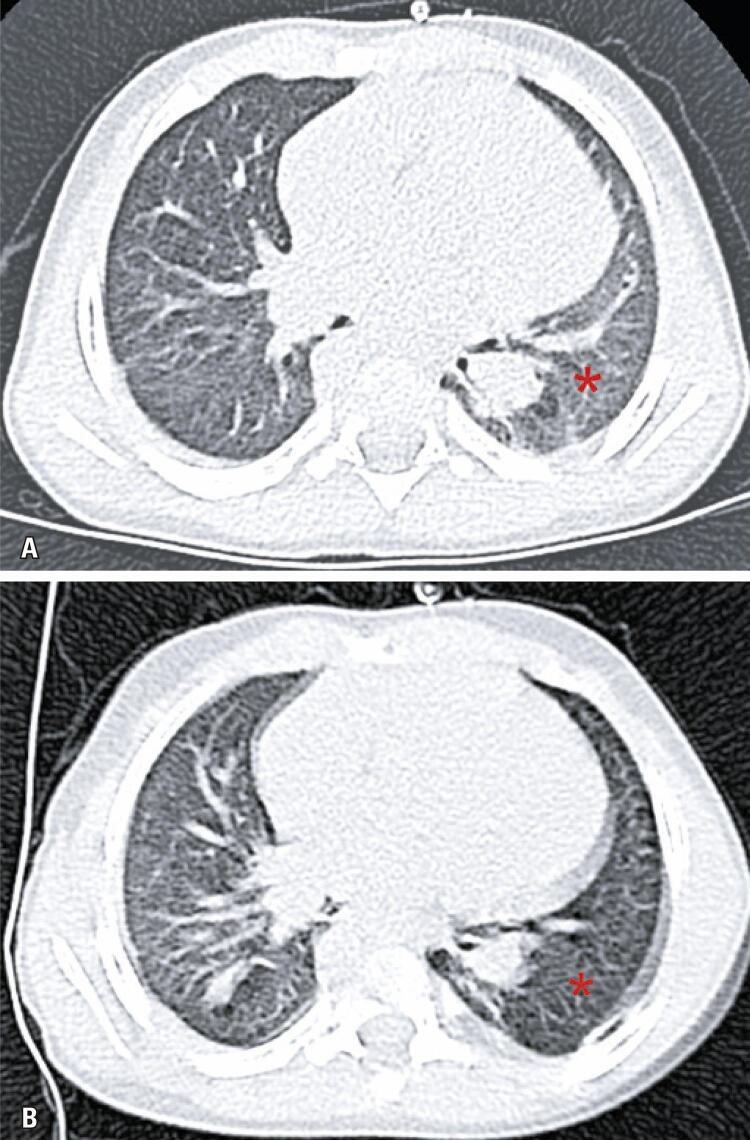
Sixteen month-old boy with suspected COVID-19 infection. (A) Increased subpleural lung attenuation of the left lower lobe suggestive of ground-glass opacities in the image acquired in the supine position; (B) Findings were not confirmed in the image acquired in right lateral decubitus. This case would be classified as negative according to Consensus and 1 according to CO-RADS; however, laboratory test (reverse transcription-polymerase chain reaction) results were positive for COVID-19

**Figure 2:**
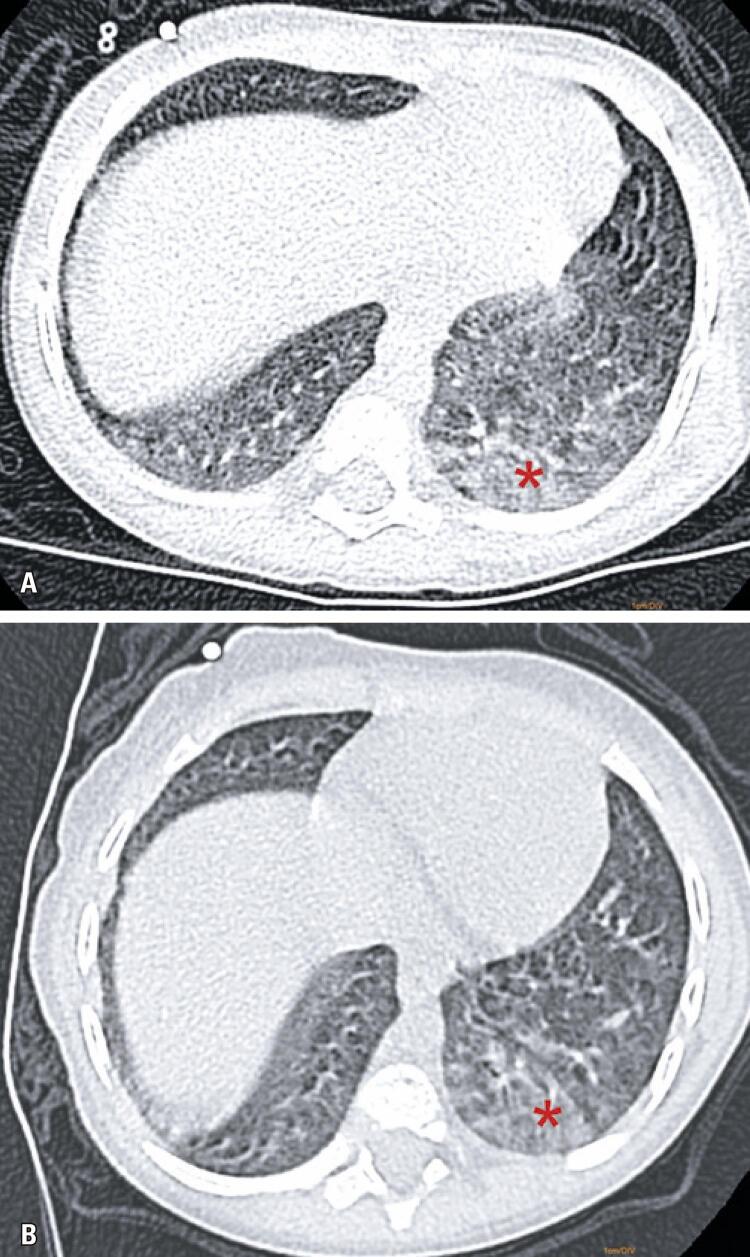
Twenty-four month-old boy with suspected COVID-19 infection. (A) Increased subpleural lung attenuation in the posterior segment of the left lower lobe suggestive of ground-glass opacities in the image acquired in the supine position; (B) Persistence of increased attenuation in the hemithorax facing upwards in the image acquired in right lateral decubitus confirmed ground-glass opacities. This case would be classified as typical according to Consensus and 5 according to CO-RADS. However, laboratory test (reverse transcription-polymerase chain reaction) results were positive for cytomegalovirus infection and negative for COVID-19

In the Lateral Decubitus Chest CT Group, ground-glass opacities were detected only when increased attenuation persisted in the upper pulmonary field of images acquired in lateral decubitus (Figures 1A and B and Figure 2A and B).

### Image interpretation

Images were independently reviewed by 2 pediatric radiologists (A and B) blinded to RT-PCR results. Imaging findings were described according to Fleischner Society guidelines^(17)^ and classified according to standardized reporting systems of CT findings in COVID-19.^(4,18)^

Standardized reporting systems were developed to facilitate the interpretation of reports by referring physicians and to stratify the risk of COVID-19.^(4,18)^ Of these, the following two systems stand out: the International Pediatric Expert Consensus (hereinafter referred to as “Consensus”) and the COVID-19 Reporting and Data System (CO-RADS).^(4,18)^The Consensus was developed for pediatric patients and classifies chest CT imaging findings as negative, atypical, indeterminate or typical of COVID-19.^(4)^ The CO-RADS was originally designed for the adult population but has also been used in children.^(18,19)^ The CO-RADS classifies chest CT imaging findings as normal or non-infectious (CO-RADS 1), typical of infections other than COVID-19 (CO-RADS 2), equivocal for COVID-19 or consistent with other viral pneumonias or non-infectious diseases (CO-RADS 3), highly suspicious of COVID-19 (CO-RADS 4) and typical COVID-19 pattern (CO-RADS 5).^(18)^

The severity of chest CT findings was semi-quantitatively reported using the chest CT score. This score was originally developed for patients with severe acute respiratory syndrome (SARS) sequelae. However it has also been used in COVID-19 patients.^(20,21)^ In this scoring system, each lobe is given a score of 0 (no pulmonary lobe involvement), 1 (<5% lobar involvement), 2 (5% to 25% lobar involvement), 3 (26% to 49% lobar involvement), 4 (50% to 75% lobar involvement) or 5 (>75% lobar involvement), the total score ranging from 0 to 25.^(20)^

The Consensus and CO-RADS classification systems were used in all patients included in this sample. The chest CT score was used in suspected cases of COVID-19 (*i.e*., Consensus = indeterminate or typical and CO-RADS = 3, 4 or 5). In the Lateral Decubitus Chest CT Group, images acquired in the supine position and in lateral decubitus were scored (*i.e.*, two scores per patient).

### Statistical analysis

Data were analyzed using SPSS 26.0 (IBM, New York, NY). Continuous variables were expressed as mean and standard deviation (SD) or median and interquartile range (IQR) (normally and not normally distributed data respectively). Associations between categorical variables were assessed using the Fisher’s exact test. Associations between continuous variables were assessed using the Student *t*-test or the Mann-Whitney U test (normally and not normally distributed variables respectively). Two-tailed p-values <0.05 were considered statistically significant.

Inter-rater reliability for CO-RADS and Consensus were calculated using the Cohen’s weighted Kappa. The strength of agreement was classified as poor (κ<0.00), slight (κ=0.21-0.40), moderate (κ=0.41-0.6), substantial (κ=0.61-0.8) or almost perfect (κ>0.80).^(22)^

Receiver operating characteristic curves were constructed for CO-RADS and Consensus. Diagnostic accuracy was classified according to the area under the curve (AUC) as failed (AUC=0.5-0.6), poor (AUC=0.6-0.7), fair (AUC=0.7-0.8), good (AUC=0.8-0.9) or excellent (AUC=0.9-1).^(23)^

Although most authors consider the chest CT score a continuous variable,^(21,24-26)^ conceptually it is an ordinal variable. Therefore, inter-rater reliability was also calculated using Cohen’s weighted Kappa and chest CT score differences between images acquired in the supine and lateral decubitus positions (Lateral Decubitus Chest CT Group) assessed using the Wilcoxon signed-rank test.

### Ethics

This single-center study was carried out in compliance with the Declaration of Helsinki and approved by the Committee on Human Research of the author’s institutions (*Hospital de Crianças* César *Pernetta* and *Hospital Pequeno Príncipe;* CAAE: 40814220.2.0000.0097; # 4.512.074). Given the retrospective nature of the study, the need for informed consent was waived.

## RESULTS

**Figure 3:**
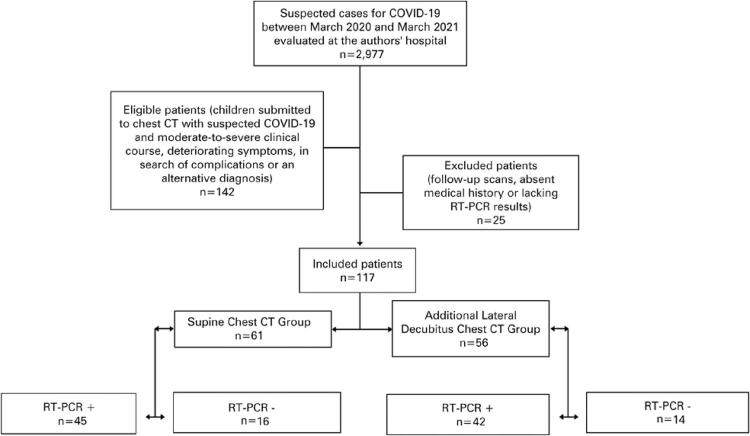
Flowchart of included and excluded patients

A total of 142 patients were submitted to chest CT during the experimental period. Of these, 25 were excluded (Figure 3). Of 117 eligible patients, 87 were positive and 30 were negative for SARS-CoV-2 on RT-PCR (74% and 26% respectively). Fifty-six patients (48%) were submitted to additional lateral decubitus chest CT image acquisition, of whom 75% were aged <2 years (Table 1).

**Table 1:** CT: computed tomography; RT-PCR: reverse transcription-polymerase chain reaction. . Demographic and clinical data

Variable	Supine Chest CT Group (n=61)	Lateral Decubitus Chest CT Group (n=56)	p value
Age (median, IQR in years)	13 (7.5-15)	1 (0.5- 2)	<0.001
Sex, n (%)	31 (51)	37 (66)	0.133
Male			
Respiratory comorbidity, any comorbidities, n (%)*	17 (28)	18 (32)	0.688
RT-PCR positive, n (%)	45 (74)	42 (75)	1

* Superimposed lung infection, acute non-infectious pulmonary disease or chronic respiratory comorbidities, including typical and atypical pneumonia, other viral pneumonia, tuberculosis, pulmonary edema, pulmonary hemorrhage, asthma, post-infectious sequelae, restrictive lung disease, bronchiolitis obliterans, childhood interstitial lung disease and pulmonary veno-occlusive disease.

IQR: interquartile range; RT-PCR: reverse transcription-polymerase chain reaction; CT: computed tomography.

Moderate to substantial inter-rater reliability was observed for Consensus and CO-RADS. However, reliability did not improve in the group submitted to additional lateral decubitus image acquisition (Table 2).

**Table 2:** Inter-rater reliability for Consensus and CO-RADS(4,18)

Correlations	Reporting system	Kappa	95%CI	p value
Supine Chest CT Group (n=61)				
Inter-rater reliability	Consensus*	0.736	0.623-0.850	<0.001
	CO-RADS	0.695	0.590-0.799	<0.001
Lateral Decubitus Chest CT Group (n=56)				
Inter-rater reliability	Consensus*	0.574	0.458-0.690	<0.001
	CO-RADS	0.679	0.543-0.815	<0.001

* International Pediatric Expert Consensus.

95%CI: 95% confidence interval; CO-RADS: COVID-19 Reporting and Data System; CT: computed tomography.

Consensus and CO-RADS failed to predict COVID-19 and no accuracy improvement was obtained in the Lateral Decubitus Chest CT Group (Table 3).

**Table 3:** Diagnostic accuracy of standardized reporting systems(4,18)

Radiologist	Reporting system	AUC	95%CI
Supine Chest CT Group (n=61)			
A	Consensus*	0.517	0.356-0.679
	CO-RADS	0.524	0.364-0.684
B	Consensus*	0.586	0.437-0.736
	CO-RADS	0.608	0.463-0.752
Lateral Decubitus Chest CT Group (n=56)			
A	Consensus*	0.512	0.340-0.684
	CO-RADS	0.491	0.314-0.667
B	Consensus*	0.553	0.393-0.713
	CO-RADS	0.529	0.373-0.685

* International Pediatric Expert Consensus.

AUC: Area under the curve; 95%CI: 95% confidence interval; CT: computed tomography; CO-RADS: COVID-19 Reporting and Data System.

Inter-rater chest CT score agreement was substantial, regardless of scanning technique (Supine Chest CT Group, weighted kappa, 0.670, 95%CI: 566-0.775, p<0.00; Lateral Decubitus Chest CT Group, weighted kappa, 0.620, 95%CI: 0.475-0.765, p<0.001). In the Lateral Decubitus Chest CT Group, chest CT scores assigned to lateral decubitus images were significantly lower compared to supine position images (Table 4).

**Table 4:** Chest computed tomography score in the Lateral Decubitus Chest Computed Tomography Group (n=56)

Radiologist	Supine position images		Lateral decubitus position images		p value
	Median	IQR	Median	IQR	
A	9	5-12	5	3-10	<0.001
B	9	7-11	8	5-11	0.003

IQR: interquartile range.

## DISCUSSION

Additional chest CT imaging in lateral decubitus does not add benefit to the diagnosis of COVID-19 in children but does increase the precision of estimated pulmonary involvement.

The diagnosis of COVID-19 is usually confirmed by RT-PCR.^(2,18)^ However, since results may take hours to days, chest CT plays a significant role in clinical work-up and management guidance.^(18)^ Although there are no specific target doses for chest CT in COVID-19, several authors endorse the use of low-dose protocols due to reduced radiation dose exposure with no significant compromise of signal-to-noise or contrast-to-noise ratios.^(27,28)^

The contribution of lateral decubitus CT imaging to the assessment of air trapping and ground-glass opacities has been well established in the literature.^(11,12)^ In these images, the hemithorax facing upwards is usually well aerated (corresponding to an inspiratory image), whereas the dependent hemithorax is poorly aerated (corresponding to an expiratory image).^(11,12)^ In images acquired in the supine position, inadequate inspiration in uncooperative patients may result in artifacts that mimic ground-glass opacities (respiratory motion or atelectasis in dependent lung segments).^(13)^ Lateral decubitus imaging can be used to distinguish between such respiratory artifacts and true ground-glass opacity.^(11)^ Despite their potential to enhance the precision of lung lesion severity estimation, additional lateral chest CT image acquisitions did not increase the diagnostic accuracy for COVID-19 in this study. Still, in the presence of limited diagnostic capacity due to significant motion artifacts in spite of patient immobilization, image acquisition in lateral decubitus using low-dose protocols is a valid alternative to sedation, since it is more cost-effective and eliminates the risks of adverse effects.^(14)^ Sedation increases scan costs and may cause respiratory depression, inability to maintain an open airway, cardiac arrest, prolonged restlessness, emergence delirium, post-sedation agitation and long-term harmful effects on the developing brain, such as behavioral and cognitive impairment.^(14,29,30)^

Findings of this study do not support the diagnostic performance of CO-RADS reported in adults. However, our results are consistent with the literature regarding CO-RADS and chest CT score inter-rater reliability. Fair to excellent diagnostic accuracy and moderate to almost perfect inter-rater agreement for CO-RADS and the chest CT score have been reported by most authors in samples comprising primarily adult patients.^(18,31-34)^ Slight to moderate inter-rater agreement regarding CT findings has also been reported in children.^(35)^ However, to the best of our knowledge, studies addressing diagnostic performance and inter-rater agreement of the Consensus classification and the CO-RADS scheme in pediatric patient populations have not been carried out to date. The low accuracy of the Consensus and CO-RADS scores for diagnosis of COVID-19 infection in children is likely due to the heterogeneous and nonspecific nature of lung CT findings in this age group.

This study has some limitations, such as retrospective design, small sample size and inclusion of only 2 independent raters. Further multi-center prospective studies with larger samples and a larger number of raters are warranted to confirm our findings.

## CONCLUSION

Additional lateral decubitus chest computed tomographic imaging does not increase diagnostic accuracy for COVID-19. The only benefit provided by lateral decubitus imaging is a more accurate estimation of lung involvement in uncooperative patients. However, this technique should not be performed routinely. In the presence of limited diagnostic capacity due to significant motion artifacts in spite of patient immobilization, lateral decubitus imaging using low-dose protocols may be a cost-effective alternative to sedation.
